# Unsteady squeezing flow of Cu-Al_2_O_3_/water hybrid nanofluid in a horizontal channel with magnetic field

**DOI:** 10.1038/s41598-021-93644-4

**Published:** 2021-07-08

**Authors:** Najiyah Safwa Khashi’ie, Iskandar Waini, Norihan Md Arifin, Ioan Pop

**Affiliations:** 1 Fakulti Teknologi Kejuruteraan Mekanikal Dan Pembuatan, Universiti Teknikal Malaysia Melaka, Hang Tuah Jaya, 76100 Durian Tunggal, Melaka, Malaysia; 2 Department of Mathematics, Faculty of Science, Universiti Putra Malaysia, 43400 UPM Serdang, Selangor, Malaysia; 3 Department of Mathematics, Babeş-Bolyai University, 400084 Cluj-Napoca, Romania

**Keywords:** Engineering, Mathematics and computing, Physics

## Abstract

The proficiency of hybrid nanofluid from Cu-Al_2_O_3_/water formation as the heat transfer coolant is numerically analyzed using the powerful and user-friendly interface bvp4c in the Matlab software. For that purpose, the Cu-Al_2_O_3_/water nanofluid flow between two parallel plates is examined where the lower plate can be deformed while the upper plate moves towards/away from the lower plate. Other considerable factors are the wall mass suction/injection and the magnetic field that applied on the lower plate. The reduced ordinary (similarity) differential equations are solved using the bvp4c application. The validation of this novel model is conducted by comparing a few of numerical values for the reduced case of viscous fluid. The results imply the potency of this heat transfer fluid which can enhance the heat transfer performance for both upper and lower plates approximately by 7.10% and 4.11%, respectively. An increase of squeezing parameter deteriorates the heat transfer coefficient by 4.28% (upper) and 5.35% (lower), accordingly. The rise of suction strength inflates the heat transfer at the lower plate while the presence of the magnetic field shows a reverse result.

## Introduction

The enrichment of heat transfer performance for the working fluid in cooling/heating appliances with optimum use of cost and energy has been the main concern for industrial and societal benefits. The addition of a single nanoparticle into a host working fluid is originated by Choi^1^ to enhance the base fluid’s thermal conductivity. Since then, many investigations were conducted through experimental works or fundamental studies (computational boundary layer flow) with less cost and time consumption. The model by Buongiorno^2^ which related to the effects of Brownian motion and thermopheresis as well as the model by Tiwari and Das^3^, are widely used by many researchers in the computational analysis of the nanofluid’s flow. Using the spectral relaxation method, Oyelakin et al.^4^ solved the Buongiorno’s model of Casson nanofluid flow with the inclusion of slip and convective conditions, magnetic field and thermal radiation. Further, Oyelakin et al.^5^ discussed the three-dimensional flow of unsteady magnetohydrodynamics (MHD) Casson nanofluid utilizing the Buongiorno’s model. Another interesting works regarding the Buongiorno’s model of nanofluid can be read from these papers^6–11^. Meanwhile, Karmakar et al.^12^ scrutinized the stagnation point flow of carbon nanotubes (CNT)-water nanofluid towards a stretching sheet with convective boundary condition. In recent times, the combination of a base fluid with suspended dissimilar nanoparticles is experimentally conducted to create a superior nanocomposite liquid known as hybrid nanofluid. The hybrid nanofluid’s correlations by Takabi and Salehi^13^ and Devi and Devi^14^ were extensively used in the estimation of the thermophysical properties. The advantages of hybrid nanofluid in augmenting the heat transfer performance could also be found in these references^15–21^. For the distant future of the cooling/heating applications, there are tremendous demands in the investigation of both internal and external hybrid nanofluid flows. Acharya et al.^22^ observed the temperature profile of working fluid with Cu-TiO_2_ nanosuspension was greater than single nanofluids when it was streaming over a revolving disk. The inclined magnetic field with a suitable inclination angle was shown as one of the potential factors in augmenting the thermal rate of the hybrid nanofluid^23^. Further assessment of the nonlinear radiation and magnetic field effects have been conducted by Acharya et al.^24^ for hybrid Ag-Fe_3_O_4_/kerosene nanofluid flow over a permeable stretching sheet. The Cu-Al_2_O_3_/water hybrid nanofluid flow past an exponentially stretching/shrinking surface was recently studied by Wahid et al.^25,26^.

The squeezing flow which emerged from the moving boundaries is significant in polymer processing, lubrication equipment, molding’s injection, and compression including the hydrodynamical machines. The connection between the squeezing flow and the loaded bearings’ performance in engines including the phenomenon of adhesion has been highlighted by Jackson^27^. Stefan^28^ used lubrication approximation to initiate his work on squeezing flow. Meanwhile, other early works considering numerical schemes on the squeezing flow were studied by Verma^29^ and Singh et al.^30^. Hamza^31^ inspected the squeezing flow and highlighted the impact of the suction and injection parameters. Other interesting papers reflecting the squeezing viscous flow have been debated in Ahmad et al.^32^, Shah et al.^33^, Khan et al.^34^, Magalakwe et al.^35^ and Basha^36^. Later, the analysis of squeezing flow in nanofluids was also considered by a few of researchers. The time-dependent bioconvection flow containing motile gyrotactic microorganisms was solved by Raees et al.^37^ using Buongiorno’s model of nanofluid. Hayat et al.^38^ analyzed the unsteady squeezed flow of nanofluid with the presence of magnetic field while Hayat et al.^39^ scrutinized the effect of couple stress due to time-dependent applied magnetic field. Both Hayat et al.^38,39^ implemented the Buongiorno nanofluid model which indirectly examined any specific nanoparticles. Recently, Acharya et al.^40^ analyzed the simultaneous effects of chemical reaction, magnetic field, and second-order slip on the bioconvection nanofluid squeezing flow between two parallel plates. Meanwhile, interesting work of the squeezing hybrid nanofluid with Fe_3_O_4_-MoS_2_ and the combination of water and ethylene glycol for the base fluid was conducted by Salehi et al.^41^. The impact of radiation from the solar energy on the Cu-Al_2_O_3_/water hybrid nanofluid inside a channel was deliberated by Acharya^42^. Another interesting aspect of the internal hybrid nanofluid flow inside a channel also has been scrutinized by Ikram et al.^43^ and Islam et al.^44^. Detail description of previous works^37–44^ concerning the internal flow between two plates is presented in Table 1 which highlights the gap between previous works and the present study.

**Table 1:** Detail description of references concerning the internal flow between two plates.

References	Single/Hybrid Nanofluid	Description of lower and upper plates	Additional physical parameters	Method of solution
Raees et al.^37^	Unsteady flow of single nanofluid (Buongiorno model)	Both lower and upper plates are impermeable	Bioconvection	Homotopy Analysis Method
Hayat et al.^38^	Unsteady flow of single nanofluid (Buongiorno model)	Lower plate is permeable and stretchable	Magnetic field	Homotopy Analysis Method
Hayat et al.^39^	Unsteady flow of single nanofluid (Buongiorno model)	Lower plate is permeable and stretchable	Magnetic field and couple stress viscosity effect	Homotopy Analysis Method
Acharya et al.^40^	Single nanofluid flow (Buongiorno model)	–	Bioconvection, magnetic field, chemical reaction and second order slip	Runge–Kutta-Fehlberg method
Salehi et al.^41^	Unsteady flow of hybrid Fe3O4-MoS2/mixture of ethylene glycol–water (correlations of hybrid nanofluid as in Devi and Devi^14^)	Lower plate is impermeable and static	Magnetic field and heat generation	Akbari and Ganji's method
Acharya^42^	Hybrid Cu-Al2O3/water (correlations of hybrid nanofluid as in Takabi and Salehi^13^)	Lower plate is stretchable Upper plate is permeable	Solar radiation	Shooting method
Ikram et al.^43^	Hybrid Ag-TiO2/water (correlations of hybrid nanofluid as in Takabi and Salehi^13^)	–	Magnetic field, natural convection and heat generation	Laplace transform method
Islam et al.^44^	Micropolar hybrid GO-Cu/water (correlations of hybrid nanofluid as in Takabi and Salehi^13^)	Lower plate is stretchable Upper plate is permeable	Magnetic field, thermal radiation and rotating system	Homotopy Analysis Method

Inspired from the existing literature while fulfilling the available research gaps, this work aims to analyze the time-dependent squeezing flow of hybrid Cu-Al2O3/water nanofluid in a horizontal channel (between two parallel plates) with the magnetic field effect. The physical geometry of the lower plate is presumed as permeable and stretchable. Our main focus is to analyze the features of hybrid nanofluid flow like distribution of velocity, skin friction, temperature, and thermal rate for several physical parameters such as suction/injection, stretching, unsteadiness squeezing, the magnetic and volumetric concentration of the nanoparticles. In long term, this study is can be applied in designing an optimum thermal process for example in refrigeration systems and heat pumps using relevant physical sources. To accomplish the objectives, the single-phase mathematical model of Cu-Al2O3/water is formulated based on this physical problem and then, transformed into reduced differential equations via the similarity transformation. The bvp4c application is completely used for the results’ generation and validated based on the available numerical values from the previous works. This study is novel and original which considers a time-dependent Cu-Al2O3/water hybrid nanofluid flow with different boundary conditions as compared to the existing references in Table 1.

## Mathematical model

### Physical assumptions and thermophysical correlations

**Figure 1:**
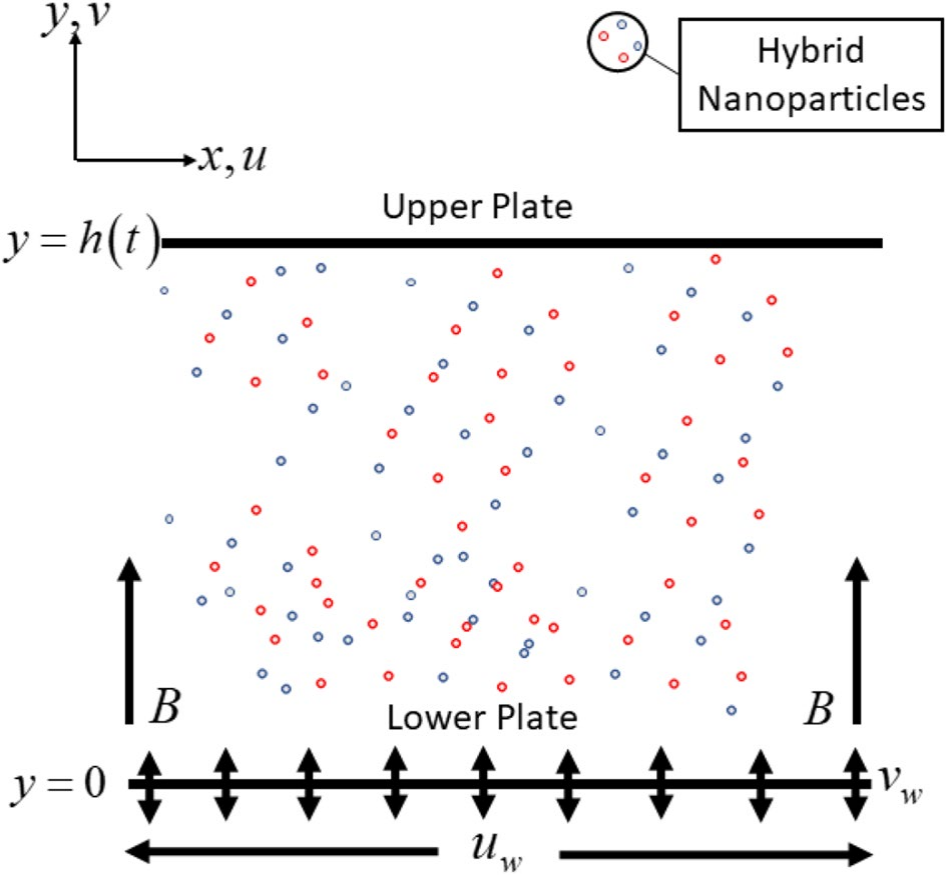
Physical illustration with coordinate system.

Cu-Al_2_O_3_/water formation is considered to flow between two infinite parallel plates, as shown in Fig. 1. The upper plate is placed at $y = h\left( t \right) = \sqrt {\frac{{\nu _{f} \left( {1 - \alpha t} \right)}}{b}}$ from the lower plate, while the upper plate with velocity $V_{h}  = \frac{{dh\left( t \right)}}{{dt}} =  - \frac{\alpha }{2}\sqrt {\frac{{\nu _{f} }}{{b\left( {1 - \alpha t} \right)}}}$ is moving towards (squeezing) the lower plate. Further assumption is the lower and upper plates are maintained at fixed temperatures $T_{1}$ and $T_{2}$, respectively. Meanwhile, the porous lower plate is included in the physical illustration for the possible fluid suction/injection with the wall mass velocity is denoted as $v_{w}  =  - \frac{{V_{0} }}{{1 - \alpha t}}$; $V_{0}  > 0$ for suction, $V_{0}  < 0$ for injection and $V_{0}  = 0$ corresponds to an impermeable plate. Also, the lower plate is stretchable with linear velocity $u_{w}  = \frac{{bx}}{{1 - \alpha t}};t < {1 \mathord{\left/ {\vphantom {1 \alpha }} \right. \kern-\nulldelimiterspace} \alpha }$ while the inclusion of time-dependent magnetic field is formulated with $B\left( t \right) = \frac{{B_{0} }}{{1 - \alpha t}}$ (see Hayat et al.^38,39^).

Under these assumptions and using the hybrid nanofluid model proposed by Takabi and Salehi^13^, the governing conservation equations are^37–39^.

1 $$ \frac{{\partial u}}{{\partial x}} + \frac{{\partial v}}{{\partial y}} = 0, $$

2 $$ \frac{{\partial V}}{{\partial t}} + u\frac{{\partial V}}{{\partial x}} + v\frac{{\partial V}}{{\partial y}} = \frac{{\mu _{{hnf}} }}{{\rho _{{hnf}} }}\frac{{\partial ^{2} V}}{{\partial y^{2} }} - \frac{{\sigma _{{hnf}} }}{{\rho _{{hnf}} }}B\left( t \right)^{2} V, $$

3 $$ \frac{{\partial T}}{{\partial t}} + u\frac{{\partial T}}{{\partial x}} + v\frac{{\partial T}}{{\partial y}} = \frac{{k_{{hnf}} }}{{(\rho C_{p} )_{{hnf}} }}\frac{{\partial ^{2} T}}{{\partial y^{2} }}, $$

where $V = \frac{{\partial v}}{{\partial x}} - \frac{{\partial u}}{{\partial y}}$. The associate conditions at the lower and upper plates are (see Raees et al.^37^ and Hayat et al.^38,39^)

4 $$ \begin{gathered}   u = \lambda \frac{{bx}}{{1 - \alpha t}},{\text{   }}v =  - \frac{{V_{0} }}{{1 - \alpha t}},{\text{   }}T = T_{1} {\text{     at     }}y = 0{\text{   (lower plate)}} \hfill \\   u = 0,{\text{   }}v = \frac{{dh\left( t \right)}}{{dt}},{\text{   }}T = T_{2} {\text{     at     }}y = h\left( t \right){\text{   (upper plate)}} \hfill \\  \end{gathered} $$

Here $u$ and $v$ are the velocities along $x$ and $y$ directions and $T$ is the hybrid nanofluid temperature. For the evaluation of the thermophysical properties (see Table 2), we adopt the correlations by Takabi and Salehi^13^ which are feasible and correct based on the experimental validation. These correlations are built based on the physical assumptions. Meanwhile, Table 3 display the the physical properties of the pure water and nanoparticles.

**Table 2:** Hybrid nanofluid’s correlations^13^.

Properties	Hybrid Nanofluid
Density $\left( \rho \right)$	$\rho _{{hnf}} = \left( {1 - \phi _{{hnf}} } \right)\rho _{f} + \phi _{1} \rho _{{s1}} + \phi _{2} \rho _{{s2}}$
Heat Capacity $\left( {\rho C_{p} } \right)$	$\left( {\rho C_{p} } \right)_{{hnf}} = \left( {1 - \phi _{{hnf}} } \right)\left( {\rho C_{p} } \right)_{f} + \phi _{1} \left( {\rho C_{p} } \right)_{{s1}} + \phi _{2} \left( {\rho C_{p} } \right)_{{s2}}$
Dynamic Viscocity $\left( \mu \right)$	$\frac{{\mu _{{hnf}} }}{{\mu _{f} }} = \frac{1}{{\left( {1 - \phi _{{hnf}} } \right)^{{2.5}} }}$
Thermal Conductivity $\left( k \right)$	$$\frac{{k_{{hnf}} }}{{k_{f} }} = \left[ {\frac{\begin{gathered} \left( {\frac{{\phi _{1} k_{1} + \phi _{2} k_{2} }}{{\phi _{{hnf}} }}} \right) + 2k_{f} + 2\left( {\phi _{1} k_{1} + \phi _{2} k_{2} } \right) \hfill \\ - 2\phi _{{hnf}} k_{f} \hfill \\ \end{gathered} }{\begin{gathered} \left( {\frac{{\phi _{1} k_{1} + \phi _{2} k_{2} }}{{\phi _{{hnf}} }}} \right) + 2k_{f} - \left( {\phi _{1} k_{1} + \phi _{2} k_{2} } \right) \hfill \\ + \phi _{{hnf}} k_{f} \hfill \\ \end{gathered} }} \right]$$
Electrical Conductivity $\left( \sigma \right)$	$$\frac{{\sigma _{{hnf}} }}{{\sigma _{f} }} = \left[ {\frac{\begin{gathered} \left( {\frac{{\phi _{1} \sigma _{1} + \phi _{2} \sigma _{2} }}{{\phi _{{hnf}} }}} \right) + 2\sigma _{f} + 2\left( {\phi _{1} \sigma _{1} + \phi _{2} \sigma _{2} } \right) \hfill \\ - 2\phi _{{hnf}} \sigma _{f} \hfill \\ \end{gathered} }{\begin{gathered} \left( {\frac{{\phi _{1} \sigma _{1} + \phi _{2} \sigma _{2} }}{{\phi _{{hnf}} }}} \right) + 2\sigma _{f} - \left( {\phi _{1} \sigma _{1} + \phi _{2} \sigma _{2} } \right) \hfill \\ + \phi _{{hnf}} \sigma _{f} \hfill \\ \end{gathered} }} \right]$$

**Table 3:** Thermophysical properties for pure water and nanoparticles^45,46^.

Thermophysical Properties	H_2_O	Nanoparticles	
		Al_2_O_3_	Cu
$\rho {\text{~}}\left( {{\text{kgm}}^{{ - 3}} } \right)$	997.1	3970	8933
$C_{p} \left( {{\text{Jkg}}^{{ - 1}} {\text{K}}^{{ - 1}} } \right)$	4179	765	385
$k{\text{~}}\left( {{\text{Wm}}^{{ - 1}} {\text{K}}^{{ - 1}} } \right)$	0.6130	40	400
$\sigma {\text{~}}\left( {sm^{{ - 1}} } \right)$	0.05	3.69 $\times 10^{7}$	5.96 $\times 10^{7}$

### Reduced differential equations

According to Raees et al.^37^ and Hayat et al.^38,39^, the suitable dimensionless variables are

5 $$ \left. \begin{gathered}   \psi  = \sqrt {\frac{{b\nu _{f} }}{{1 - \alpha t}}} xf\left( \eta  \right),{\text{   }}u = \frac{{bx}}{{1 - \alpha t}}f^{\prime}\left( \eta  \right),{\text{   }}v =  - \sqrt {\frac{{b\nu _{f} }}{{1 - \alpha t}}} f\left( \eta  \right),{\text{   }} \hfill \\   \eta  = \sqrt {\frac{b}{{\nu _{f} \left( {1 - \alpha t} \right)}}} y,{\text{   }}\theta \left( \eta  \right) = \frac{{T - T_{0} }}{{T_{2}  - T_{0} }} \hfill \\  \end{gathered}  \right\} $$

Hence, substituting (5) into Eqs. (2)-(4), the ODEs and boundary conditions are

6 $$ \left( {\frac{{{{\mu _{{hnf}} } \mathord{\left/ {\vphantom {{\mu _{{hnf}} } {\mu _{f} }}} \right. \kern-\nulldelimiterspace} {\mu _{f} }}}}{{{{\rho _{{hnf}} } \mathord{\left/ {\vphantom {{\rho _{{hnf}} } {\rho _{f} }}} \right. \kern-\nulldelimiterspace} {\rho _{f} }}}}} \right)f^{{iv}}  + ff^{\prime\prime\prime} - f^{\prime}f^{\prime\prime} - \frac{{Sq}}{2}\left( {3f^{\prime\prime} + \eta f^{\prime\prime\prime}} \right) - \left( {\frac{{{{\sigma _{{hnf}} } \mathord{\left/ {\vphantom {{\sigma _{{hnf}} } {\sigma _{f} }}} \right. \kern-\nulldelimiterspace} {\sigma _{f} }}}}{{{{\rho _{{hnf}} } \mathord{\left/ {\vphantom {{\rho _{{hnf}} } {\rho _{f} }}} \right. \kern-\nulldelimiterspace} {\rho _{f} }}}}} \right)Mf^{\prime\prime} = 0, $$

7 $$ \frac{1}{{\Pr }}\frac{{{{k_{{hnf}} } \mathord{\left/ {\vphantom {{k_{{hnf}} } {k_{f} }}} \right. \kern-\nulldelimiterspace} {k_{f} }}}}{{{{\left( {\rho C_{p} } \right)_{{hnf}} } \mathord{\left/ {\vphantom {{\left( {\rho C_{p} } \right)_{{hnf}} } {\left( {\rho C_{p} } \right)_{f} }}} \right. \kern-\nulldelimiterspace} {\left( {\rho C_{p} } \right)_{f} }}}}\theta ^{\prime\prime} + f\theta ^{\prime} - \frac{{Sq}}{2}\eta \theta ^{\prime} = 0, $$

8 $$ \begin{gathered}   f^{\prime}\left( 0 \right) = \lambda ,{\text{   }}f\left( 0 \right) = S,{\text{   }}\theta \left( 0 \right) = \delta , \hfill \\   f^{\prime}\left( 1 \right) = 0,{\text{   }}f\left( 1 \right) = \frac{{Sq}}{2},{\text{   }}\theta \left( 1 \right) = 1. \hfill \\  \end{gathered} $$

where $\lambda  > 0$ refers to the stretching lower plate and $\lambda  = 0$ denotes the fixed/static lower plate. Another dimensionless parameters are the unsteadiness squeezing parameter $Sq$, magnetic parameter $M$, suction parameter $S$, Prandtl number $\Pr$ and constant $\delta$. These parameters are defined as^37–39^

9 $$ \Pr  = \frac{{\left( {\rho C_{p} } \right)_{f} }}{{k_{f} }},{\text{     }}Sq = \frac{\alpha }{b},{\text{     }}M = \frac{{\sigma _{f} B_{0} }}{{b\rho _{f} }},{\text{     }}S = \frac{{V_{0} }}{{bh}},{\text{     }}\delta  = \frac{{T_{1}  - T_{0} }}{{T_{2}  - T_{0} }},{\text{     }} $$

In this work, we set $\delta  = 0$ which in accordance with Hayat et al.^38,39^. Further, we notice that Eq. (6) is compatible with the reduced momentum equation in Hayat et al.^38^ and Hayat et al.^39^ (if the couple stress parameter is zero) with the exclusion of the hybrid nanoparticles or $\phi _{1} ,\phi _{2}  \approx 0$ (regular fluid). The reduced skin friction coefficients and local Nusselt numbers at lower and upper plates are^37–39^

10 $$ {\text{Lower:   }}\text{Re} _{x} ^{{1/2}} C_{{f1}}  = \frac{{\mu _{{hnf}} }}{{\mu _{f} }}f^{\prime\prime}\left( 0 \right){\text{,              Upper:   }}\text{Re} _{x} ^{{1/2}} C_{{f2}}  = \frac{{\mu _{{hnf}} }}{{\mu _{f} }}f^{\prime\prime}\left( 1 \right){\text{,              }} $$

11 $$ {\text{Lower:   }}\text{Re} _{x} ^{{ - 1/2}} Nu_{{x1}}  =  - \frac{{k_{{hnf}} }}{{k_{f} }}\theta ^{\prime}\left( 0 \right){\text{,              }}\text{Re} _{x} ^{{ - 1/2}} Nu_{{x2}}  =  - \frac{{k_{{hnf}} }}{{k_{f} }}\theta ^{\prime}\left( 1 \right){\text{,              }} $$

where $$\text{Re} _{x}  = \frac{{xU_{w} }}{{\nu _{f} }}.$$

### Numerical methods and validation test

In solving the boundary layer equations, there are many methods proposed by the researchers such as homotopy analysis method (HAM), shooting technique, Keller-box method, Runge–Kutta method, Laplace transform and many others. A concise review of the numerical methods which used to solve the boundary layer equations specifically for Casson fluid was discussed by Verma and Mondal^47^. Meanwhile, Rai and Mondal^48^ reviewed spectral methods like spectral relaxation method, spectral homotopy analysis method, spectral quasi-linearization method and spectral local linearization method in solving fluid flow problem. Another interesting technique namely multi domain bivariate quasi-linearization method was used by Oyelakin et al.^49^ in solving mixed convection flow of Casson nanofluid. Meanwhile, the bvp4c solver procurable in the Matlab software was also widely used by many researchers to solve the nonlinear ODEs. It is validated that the results of limiting cases using bvp4c is in accordance with the previously published results that used another methods (i.e., analytical, shooting, Keller-box method). The finite difference method under subclass 3-stage Lobatto IIIa scheme was programmed into the bvp4c solver through a general syntax *sol* = *bvp4c (@OdeBVP, @OdeBC, solinit, options)*. For the completion of the numerical solutions in this study, Eqs. (6) to (8) are solved by transforming it first into the language of the bvp4c code as follows:

12 $$ f = y1,{\text{     }}f^{\prime} = y2,{\text{     }}f^{\prime\prime} = y3,{\text{     }}f^{\prime\prime\prime} = y4,{\text{    }} $$

13 $$ \begin{gathered}   f^{{iv}}  = \left( {\frac{{{{\rho _{{hnf}} } \mathord{\left/ {\vphantom {{\rho _{{hnf}} } {\rho _{f} }}} \right. \kern-\nulldelimiterspace} {\rho _{f} }}}}{{{{\mu _{{hnf}} } \mathord{\left/ {\vphantom {{\mu _{{hnf}} } {\mu _{f} }}} \right. \kern-\nulldelimiterspace} {\mu _{f} }}}}} \right)\left( {f^{\prime}f^{\prime\prime} - ff^{\prime\prime\prime} + \frac{{Sq}}{2}\left( {3f^{\prime\prime} + \eta f^{\prime\prime\prime}} \right) + \left( {\frac{{{{\sigma _{{hnf}} } \mathord{\left/ {\vphantom {{\sigma _{{hnf}} } {\sigma _{f} }}} \right. \kern-\nulldelimiterspace} {\sigma _{f} }}}}{{{{\rho _{{hnf}} } \mathord{\left/ {\vphantom {{\rho _{{hnf}} } {\rho _{f} }}} \right. \kern-\nulldelimiterspace} {\rho _{f} }}}}} \right)Mf^{\prime\prime}} \right), \hfill \\   {\text{     }} = \left( {\frac{{{{\rho _{{hnf}} } \mathord{\left/ {\vphantom {{\rho _{{hnf}} } {\rho _{f} }}} \right. \kern-\nulldelimiterspace} {\rho _{f} }}}}{{{{\mu _{{hnf}} } \mathord{\left/ {\vphantom {{\mu _{{hnf}} } {\mu _{f} }}} \right. \kern-\nulldelimiterspace} {\mu _{f} }}}}} \right)\left( {y2y3 - y1y4 + \frac{{Sq}}{2}\left( {3y3 + \eta y4} \right) + \left( {\frac{{{{\sigma _{{hnf}} } \mathord{\left/ {\vphantom {{\sigma _{{hnf}} } {\sigma _{f} }}} \right. \kern-\nulldelimiterspace} {\sigma _{f} }}}}{{{{\rho _{{hnf}} } \mathord{\left/ {\vphantom {{\rho _{{hnf}} } {\rho _{f} }}} \right. \kern-\nulldelimiterspace} {\rho _{f} }}}}} \right)My3} \right), \hfill \\  \end{gathered} $$

14 $$ \theta  = y5,{\text{     }}\theta ^{\prime} = y6,{\text{    }} $$

15 $$ \begin{gathered}   \theta ^{\prime\prime} = \Pr \left( {\frac{{{{\left( {\rho C_{p} } \right)_{{hnf}} } \mathord{\left/ {\vphantom {{\left( {\rho C_{p} } \right)_{{hnf}} } {\left( {\rho C_{p} } \right)_{f} }}} \right. \kern-\nulldelimiterspace} {\left( {\rho C_{p} } \right)_{f} }}}}{{{{k_{{hnf}} } \mathord{\left/ {\vphantom {{k_{{hnf}} } {k_{f} }}} \right. \kern-\nulldelimiterspace} {k_{f} }}}}} \right)\left( {\frac{{Sq}}{2}\eta \theta ^{\prime} - f\theta ^{\prime}} \right), \hfill \\   {\text{   }} = \Pr \left( {\frac{{{{\left( {\rho C_{p} } \right)_{{hnf}} } \mathord{\left/ {\vphantom {{\left( {\rho C_{p} } \right)_{{hnf}} } {\left( {\rho C_{p} } \right)_{f} }}} \right. \kern-\nulldelimiterspace} {\left( {\rho C_{p} } \right)_{f} }}}}{{{{k_{{hnf}} } \mathord{\left/ {\vphantom {{k_{{hnf}} } {k_{f} }}} \right. \kern-\nulldelimiterspace} {k_{f} }}}}} \right)\left( {\frac{{Sq}}{2}\eta y6 - y1y6} \right), \hfill \\  \end{gathered} $$

16 $$ \begin{gathered}   f^{\prime}\left( 0 \right) = \lambda ,{\text{   }}f\left( 0 \right) = S,{\text{   }}\theta \left( 0 \right) = \delta , \hfill \\   f^{\prime}\left( 1 \right) = 0,{\text{   }}f\left( 1 \right) = \frac{{Sq}}{2},{\text{   }}\theta \left( 1 \right) = 1, \hfill \\    \hfill \\   ya2 - \lambda ,{\text{   }}ya1 - S,{\text{   }}ya5 - \delta , \hfill \\   yb2,{\text{   }}yb1 - \frac{{Sq}}{2},{\text{   }}yb5 - 1. \hfill \\  \end{gathered} $$

where $ya$ and $yb$ implies the boundary conditions at lower and upper plates, respectively. The bvp4c solver will code Eqs. (13) and (15) into @OdeBVP while the condition (16) is coded into @OdeBC. Generally, the *solinit* function refers to the initial mesh point and guesses at the mesh points. However, modifications are necessary for the *solinit* and *options* functions in the bvp4c syntax to solve the present internal flow problem which affirms the novelty of this work. The asymptotical profile (for usual boundary layer flow) is necessary when the problem is dealing with the external flow over an infinite surface where these profiles must satisfy the free stream condition. However, in this work, the validation part is based on the comparison with previous similar works as presented in Tables 4 and 5. The validation is important to highlight the precision of the present model and code. Hence, the numerical values are compared with Hayat et al.^38,39^ (main references) as displayed in Tables 4 and 5 which shows identical results when suction and magnetic parameters are considered.

**Table 4:** Comparative values of $f^{\prime\prime}\left( 0 \right)$-lower plate and $f^{\prime\prime}\left( 1 \right)$-upper plate when $Sq = 0$, $\lambda  = 1$, $\phi _{1} ,\phi _{2}  = 0$ with various $S$ and $M$.

$M$	$S$	$f^{\prime\prime}\left( 0 \right)$		$f^{\prime\prime}\left( 1 \right)$	
		Present	Hayat et al.^39^	Present	Hayat et al.^39^
0	0.5	−7.4111525	−7.411153	4.7133028	4.713303
1	0.5	−7.5916177	−7.591618	4.7390165	4.739017
4	0.5	−8.1103342	−8.110334	4.8202511	4.820251
9	0.5	−8.9100956	−8.910096	4.9648698	4.964870
4	0.0	−4.5878911	−4.587891	1.8424469	1.842447
4	0.3	−6.6656620	−6.665662	3.6536948	3.653695
4	0.6	−8.8514442	−8.851444	5.3912475	5.391248
4	1.0	−11.9485843	−11.948584	7.5934262	7.593426

**Table 5:** Comparative values of $f^{\prime\prime}\left( 1 \right)$-upper plate when $\lambda  = 1$ and $\phi _{1} ,\phi _{2}  \approx 0$ with various $S$, $Sq$ and $M$.

$M$	$Sq$	$S$	$f^{\prime\prime}\left( 1 \right)$	
			Present	Hayat et al.^38^
0	1	0.5	1.814634	1.81463
0.25	1	0	-1.171551	-1.17155
0.25	1	0.5	1.808177	1.80818
0.25	0	0.5	4.719656	4.79166
0.25	1.5	0.5	0.283948	0.28395
0.25	1	1	4.573016	4.57302
1	1	0.5	1.789372	1.78937

## Results and discussion

The results are generated and graphically presented for the distribution of the skin friction coefficients, velocity, heat transfer rates, and temperature of both upper and lower plates. The value of the Prandtl number is fixed to 6.2 which indicates the use of water at 25 °C while the initial temperature at the lower plate’s wall is represented by $\delta  = 0$ so that $\theta \left( 0 \right) = 0$. Other parameters are controlled within the ranges of $0 \le Sq \le 1$ (unsteadiness squeezing parameter), $- 1.2 \le S \le 1.2$ (suction/injection parameter), $0 \le \lambda  \le 2$ (stretching parameter), and $0 \le M \le 3$ (magnetic parameter).

Table 6 shows the variety of $\text{Re} _{x} ^{{1/2}} C_{{f1}}$, $\text{Re} _{x} ^{{1/2}} C_{{f2}}$, $- \text{Re} _{x} ^{{ - 1/2}} Nu_{{x1}}$ and $- \text{Re} _{x} ^{{ - 1/2}} Nu_{{x2}}$. With the consideration of viscous fluid, lower static plate, and the exclusion of magnetic, suction/injection, and squeezing parameters, the internal flow has zero frictions and equal heat transfer rates at both plates. The observation in Table 6 shows that as the stretching parameter increases from $\lambda  = 0$ to $\lambda  = 1$, $- \text{Re} _{x} ^{{ - 1/2}} Nu_{{x1}}$ increases, but the stretching lower plate tends to increase $\text{Re} _{x} ^{{1/2}} C_{{f1}}$. Further, we analyze four types of fluids: viscous/water $\left( {\phi _{1} ,\phi _{2}  = 0} \right)$, Al_2_O_3_-water $\left( {\phi _{1}  = 0.01,\phi _{2}  = 0} \right)$, Cu-water $\left( {\phi _{1}  = 0,\phi _{2}  = 0.01} \right)$ , and Cu-Al_2_O_3_/water $\left( {\phi _{1} ,\phi _{2}  = 0.01} \right)$ which reveals that the Cu-Al_2_O_3_/water has the highest heat transfer coefficients at both plates followed by the Cu-water, Al_2_O_3_-water, and water. This implies the suitability of Cu-Al_2_O_3_/water hybrid nanofluid as an effective coolant in engineering and technology appliances. Since the inclusion of the squeezing parameter can reduce the heat transfer rate at both plates, it is useful to know the exact parameters which can assist the heat transfer performance for this situation. In Table 7, we analyze the difference percentage of the heat transfer rate at both upper and lower plate which can be used for future assessment by other engineers or researchers. This analysis also can help in determining the preferable strength of the parameters either in the augmentation or reduction of the heat transfer in both locations.

**Table 6:** Numerical values of $\text{Re} _{x} ^{{1/2}} C_{{f1}}$, $\text{Re} _{x} ^{{1/2}} C_{{f2}}$, $- \text{Re} _{x} ^{{ - 1/2}} Nu_{{x1}}$ and $- \text{Re} _{x} ^{{ - 1/2}} Nu_{{x2}}$ with various values of the control parameters.

$Sq$	$M$	$S$	$\phi _{1}$	$\phi _{2}$	$\lambda$	$\text{Re} _{x} ^{{1/2}} C_{{f1}}$	$\text{Re} _{x} ^{{1/2}} C_{{f2}}$	$- \text{Re} _{x} ^{{ - 1/2}} Nu_{{x1}}$	$- \text{Re} _{x} ^{{ - 1/2}} Nu_{{x2}}$
0	0	0	0	0	0	0	0	1	1
0	0	0	0	0	0.5	-2.021410	0.988195	1.161853	0.898716
0	0	0	0	0	1	-4.085563	1.953179	1.336614	0.802165
0	0	0	0.01	0	1	-4.189890	2.002674	1.363747	0.831425
0	0	0	0	0.01	1	-4.194140	2.000385	1.365114	0.832586
0	0	0	0.01	0.01	1	-4.302130	2.051712	1.393870	0.863450
1	0	0	0.01	0.01	1	-1.240570	-1.220273	1.323125	0.828030
1	1	0	0.01	0.01	1	-1.322328	-1.301313	1.318921	0.831390
1	1	0.2	0.01	0.01	1	-2.688739	-0.003914	1.893656	0.668863
1	1	-0.2	0.01	0.01	1	-0.003540	-2.635965	0.887339	1.001950

**Table 7:** Heat transfer analysis with the addition of the control parameters.

Parameters	Develop/reduce the thermal rate at lower plate	Difference percentage of $- \text{Re} _{x} ^{{ - 1/2}} Nu_{{x1}}$	Develop/reduce the thermal rate at upper plate	Difference percentage of $- \text{Re} _{x} ^{{ - 1/2}} Nu_{{x2}}$
Squeezing $\left( {Sq} \right)$	Reduce	-5.35%	Reduce	-4.28%
Magnetic $\left( M \right)$	Reduce	-0.32%	Develop	0.40%
Suction $\left( {S > 0} \right)$	Develop	30.35%	Reduce	-24.30%
Injection $\left( {S < 0} \right)$	Reduce	-48.64%	Develop	17.02%

**Figure 2:**
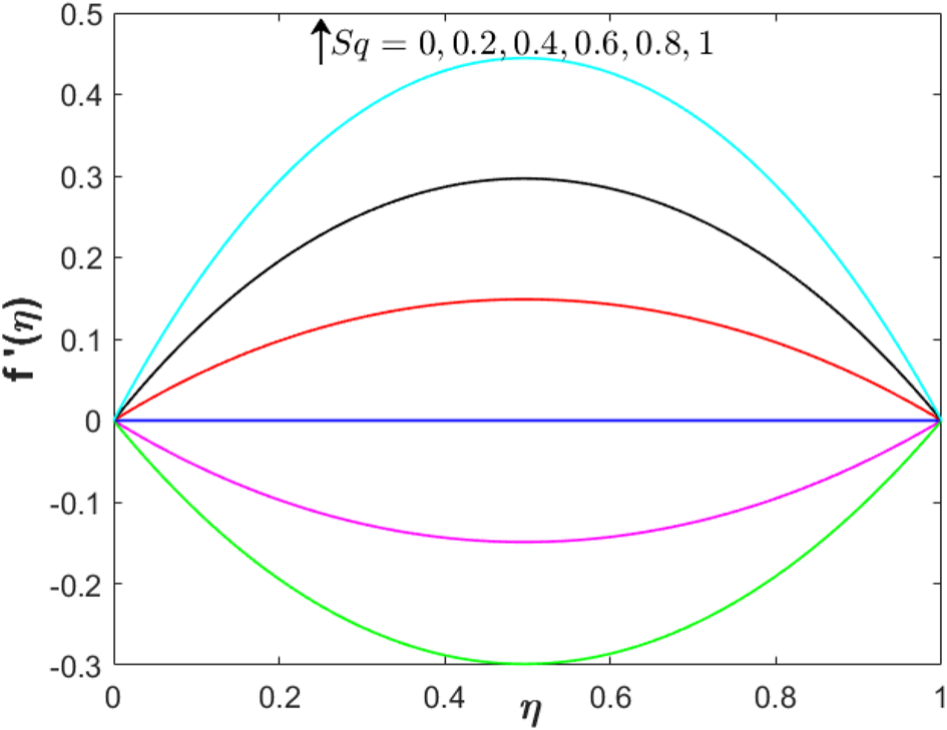
Effect of squeezing parameter on $f^{\prime}\left( \eta  \right)$ when $S = 0.2$, $\lambda  = 0$, $M = 1$ and $\phi _{1}  = \phi _{2}  = 0.01$.

**Figure 3:**
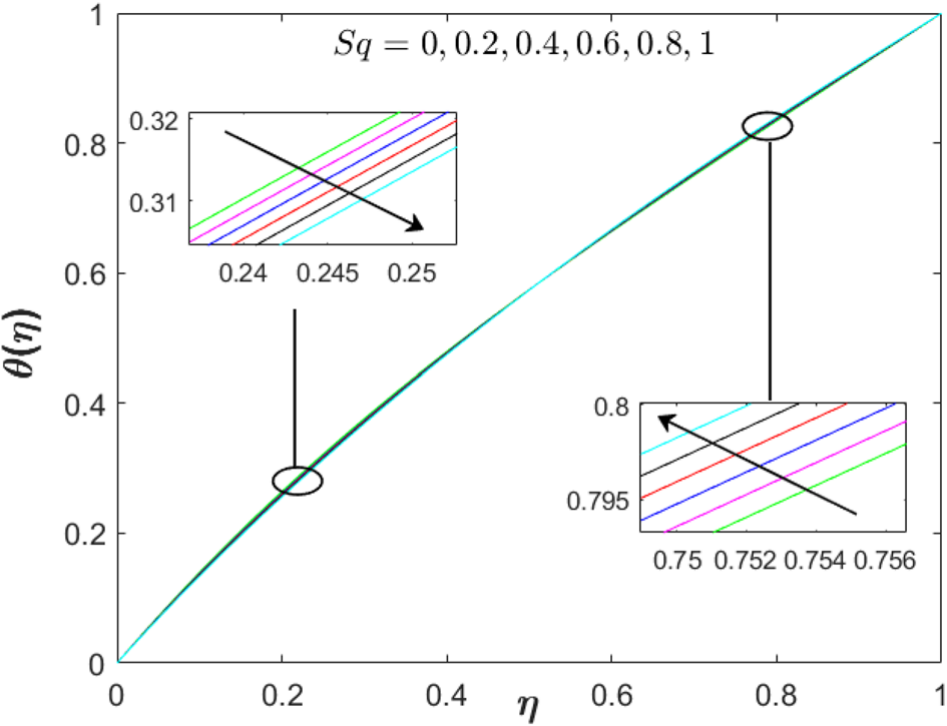
Effect of squeezing parameter on $\theta \left( \eta  \right)$ when $S = 0.2$, $\lambda  = 0$, $Sq = M = 1$ and $\phi _{1}  = \phi _{2}  = 0.01$.

**Figure 4:**
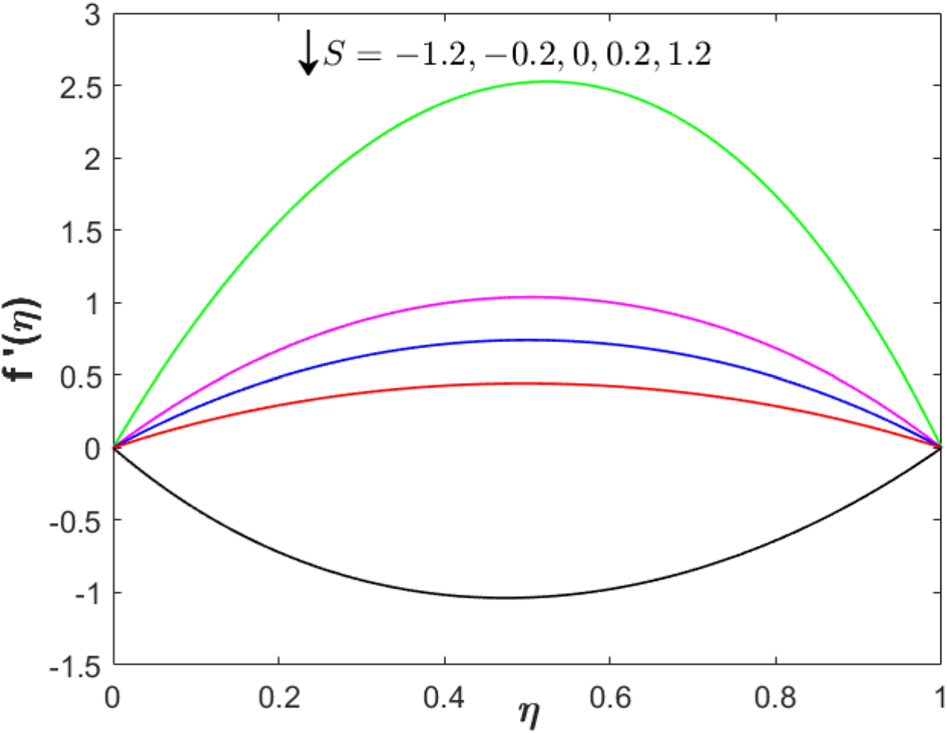
Effect of suction/injection parameter on $f^{\prime}\left( \eta  \right)$ when $\lambda  = 0$, $Sq = M = 1$ and $\phi _{1}  = \phi _{2}  = 0.01$.

**Figure 5:**
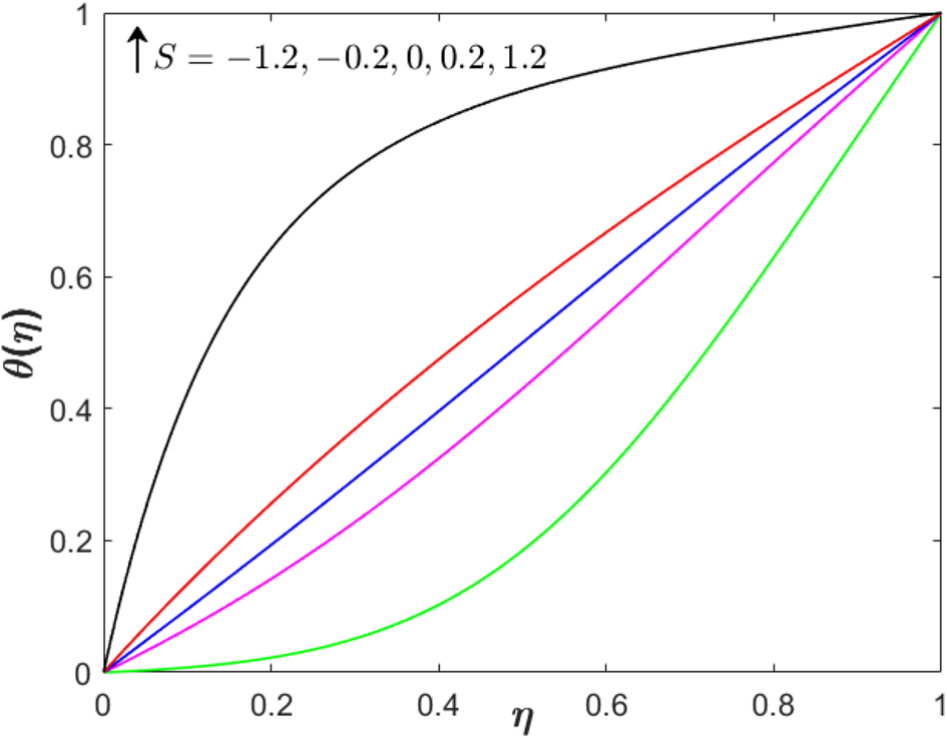
Effect of suction/injection parameter on $\theta \left( \eta  \right)$ when $\lambda  = 0$, $Sq = M = 1$ and $\phi _{1}  = \phi _{2}  = 0.01$.

**Figure 6:**
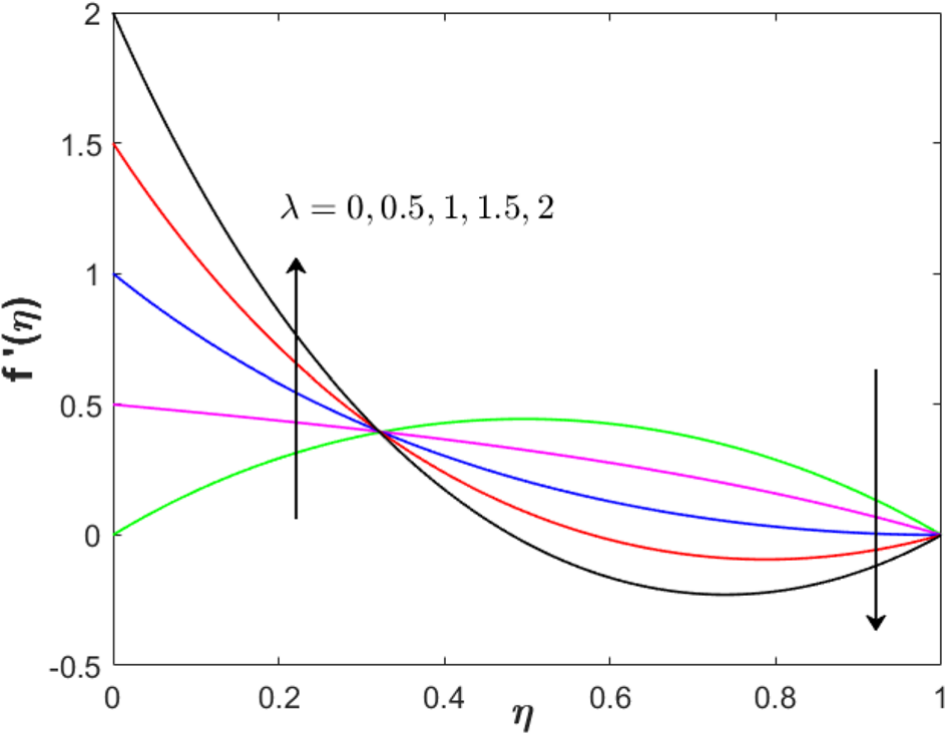
Effect of stretching parameter on $f^{\prime}\left( \eta  \right)$ when $S = 0.2$, $Sq = M = 1$ and $\phi _{1}  = \phi _{2}  = 0.01$.

**Figure 7:**
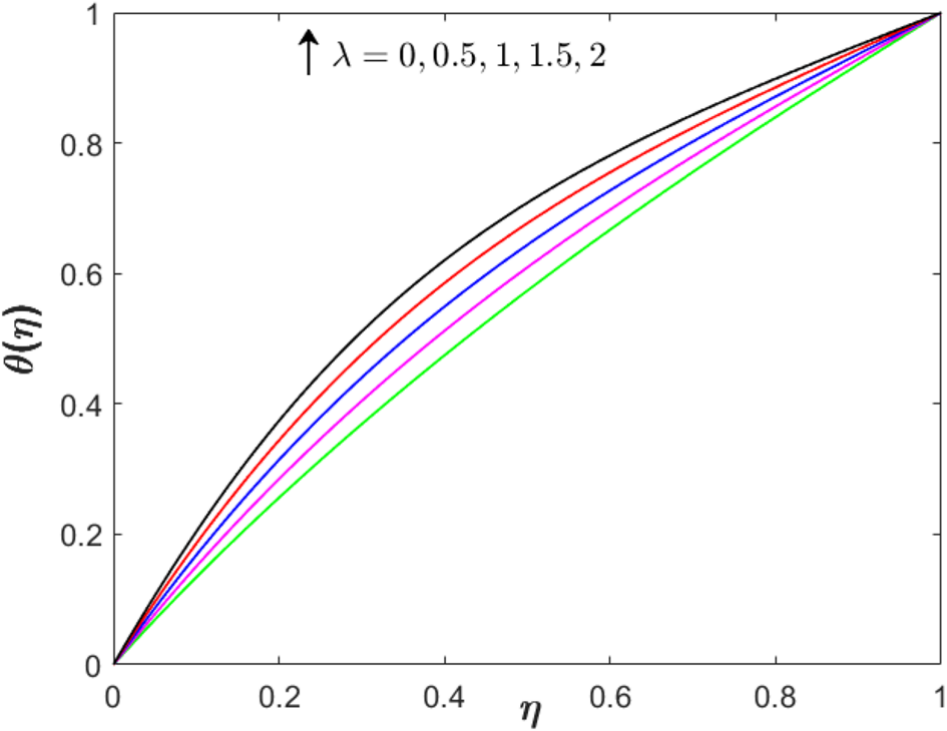
Effect of stretching parameter on $\theta \left( \eta  \right)$ when $S = 0.2$, $Sq = M = 1$ and $\phi _{1}  = \phi _{2}  = 0.01$.

The exploration of pertinent parameters’ impact such as squeezing, suction/injection, and stretching parameters is continued with the observation on $f^{\prime}\left( \eta  \right)$ and $\theta \left( \eta  \right)$ as displayed in Figs. 2–7. In Fig. 2, the velocity distribution enhances with the addition of the unsteadiness squeezing parameter. As the squeezing parameter’s magnitude increases up to $Sq = 1$, the velocity distribution is depreciated at the upper plate. This is due to the squeezing effect which originated from the upper plate. However, the temperature profile in Fig. 3 slightly reduces near to the lower plate $\left( {\eta  < 0.5} \right)$ while increases near to the upper plate $\left( {\eta  > 0.5} \right)$.

Further, the impact of the suction/injection parameter on both profiles is visualized in Figs. 4 and 5. As the suction/injection parameter increases from injection to suction $\left( {S =  - 1.2, - 0.2,0,0.2,1.2} \right)$, the velocity profile decreases which reflects the higher magnitude of suction strength can reduce the velocity distribution. Since the suction is applied through the lower permeable plate, the velocity lessens while increases near to the upper plate. The temperature profile augments at both locations (lower and upper plate). Figures 6 and 7 present the plots of velocity and temperature distribution with variety values of the stretching parameter. The velocity gradually increases when $\eta  < 0.3$ while a contrary observation is obtained for $\eta  > 0.3$. The temperature profile increases for both lower and upper plates.

## Conclusion

This novel work presents a numerical study of Cu-Al_2_O_3_/water inside two plates (parallel lower and upper) with the appearance of the magnetic field. The lower plate is permeable for the suction/injection processes and also can be stretched. Meanwhile, the upper plate can move towards the lower plate and creates the squeezing flow phenomenon. The mathematical model which suits this physical phenomenon follows the usual approximations of boundary layer flow while the bvp4c programme is fully utilized for the generation of the results. The distribution of $\text{Re} _{x} ^{{1/2}} C_{{f1}}$, $\text{Re} _{x} ^{{1/2}} C_{{f2}}$, $- \text{Re} _{x} ^{{ - 1/2}} Nu_{{x1}}$ and $- \text{Re} _{x} ^{{ - 1/2}} Nu_{{x2}}$ are examined. The heat transfer rate of the lower plate reduces with the increase of magnetic, squeezing, and injection parameters. However, about 30.35% of $- \text{Re} _{x} ^{{ - 1/2}} Nu_{{x1}}$ is developed with the inclusion of suction. Meanwhile, the enhancement of magnetic and injection parameters can lead to the development of the upper plate’s heat transfer performance. Another observation is conducted for the distribution of the velocity and temperature profiles. The addition of squeezing and stretching parameters can increase the velocity profile whereas high suction’s magnitude shows the opposite trend.

## Abbreviations

$$b$$: Constant
$B$: Time-dependent magnetic field
$$B_{0}$$: Constant
$C_{{f1}} ,C_{{f2}}$: Skin friction coefficients of lower and upper plates, respectively
$$C_{p}$$: Specific heat ($${\text{Jkg}}^{{ - 1}} {\text{K}}^{{ - 1}}$$)
$f$ (subscript): Base fluid
$$f\left( \eta \right)$$: Stream function
$hnf$(subscript): Hybrid nanofluid
$h\left( t \right)$: Distance between two plates (${\text{m}}$)
$$k$$: Thermal conductivity ($${\text{Wm}}^{{ - 1}} {\text{K}}^{{ - 1}}$$)
$$M$$: Magnetic parameter
$Nu_{{x1}} ,Nu_{{x2}}$: Local Nusselt number of lower and upper plates, respectively
$$nf$$(subscript): Nanofluid
$$\Pr$$: Prandtl number
$$t$$: Time
$T$: Hybrid nanofluid temperature (${\text{K}}$)
$T_{1} ,T_{2}$: Fixed temperatures of lower and upper plates, respectively (${\text{K}}$)
$u,v$: Fluid velocities, respectively (${\text{ms}}^{{ - 1}}$)
$u_{w}$: Velocity of the stretching lower plate (${\text{ms}}^{{ - 1}}$)
$v_{w}$: Velocity of the wall mass porous lower plate (${\text{ms}}^{{ - 1}}$)
$$V_{0}$$: Constant
$V_{h}$: Velocity of the upper plate moving towards/away from lower stationery plate (${\text{ms}}^{{ - 1}}$)
$$S$$: Suction parameter
$Sq$: Unsteadiness squeezing parameter
$s1,s2$(subscript): Al_2_O_3_ and Cu nanoparticles
$$x,y$$: Cartesian coordinates (m

## Greek symbols

$\phi _{1} ,\phi _{2}$: Concentrations for the nanoparticles
$$\eta$$: Similarity variable
$$\lambda$$: Stretching parameter
$$\mu$$: Dynamic viscosity ($${\text{kgm}}^{{ - 1}} {\text{s}}^{{ - 1}}$$)
$$\nu$$: Kinematic viscosity ($${\text{m}}^{2} {\text{s}}^{{ - 1}}$$)
$\theta$: Dimensionless fluid temperature
$$\rho$$: Fluid density ($${\text{kgm}}^{{ - 3}}$$)
$$\rho C_{p}$$: Heat capacity ($${\text{JK}}^{{ - 1}} {\text{m}}^{{ - 3}}$$

## References

[1] Choi, S.U.S. Enhancing thermal conductivity of fluids with nanoparticle. *Proc. ASME Int. Mech. Eng. Cong. Expo.* FED 231/MD66, 99–105 (1995).

[2] Buongiorno J (2006). Convective transport in nanofluids. J. Heat Transf..

[3] Tiwari RK, Das MK (2007). Heat transfer augmentation in a two-sided lid-driven differentially heated square cavity utilizing nanofluids. Int. J. Heat Mass Transf..

[4] Oyelakin IS, Mondal S, Sibanda P (2016). Unsteady Casson nanofluid flow over a stretching sheet with thermal radiation, convective and slip boundary conditions. Alexandria Eng. J..

[5] Oyelakin IS, Mondal S, Sibanda P (2017). Unsteady MHD three-dimensional Casson nanofluid flow over a porous linear stretching sheet with slip condition. Front. Heat Mass Transf..

[6] Oyelakin IS, Mondal S, Sibanda P, Sibanda D (2019). Bioconvection in Casson nanofluid flow with gyrotactic microorganisms and variable surface heat flux. Int. J. Biomath..

[7] Khashi’ie, N.S., Arifin, N.M., Hafidzuddin, E.H. & Wahi, N. Dual stratified nanofluid flow past a permeable shrinking/stretching sheet using a non-Fourier energy model. *Appl. Sci.* **9**(10), 2124 (2019).

[8] Oyelakin IS, Lalramneihmawii PC, Mondal S, Sibanda P (2020). Analysis of double-diffusion convection on three-dimensional MHD stagnation point flow of a tangent hyperbolic Casson nanofluid. Int. J. Ambient Energy.

[9] Oyelakin IS, Mondal S, Sibanda P (2020). Nonlinear radiation in bioconvective Casson nanofluid flow. Int. J. Appl. Comp. Math..

[10] Oyelakin IS, Lalramneihmawii PC, Mondal S, Nandy SK, Sibanda P (2020). Thermophysical analysis of three-dimensional magnetohydrodynamic flow of a tangent hyperbolic nanofluid. Eng. Rep..

[11] Mburu ZM, Mondal S, Sibanda P, Sharma R (2021). A numerical study of entropy generation on an oldroyd-B nanofluid flow past a riga plate. J. Therm. Eng..

[12] Karmakar S, Mpendulo MV, Mondal S (2021). Rheological analysis of CNT suspended nanofluid with convective boundary condition using spectral method. Nanosci. Nanotech. Asia.

[13] Takabi, B. & Salehi, S. Augmentation of the heat transfer performance of a sinusoidal corrugated enclosure by employing hybrid nanofluid. *Adv. Mech. Eng.* **6**, 147059 (2014).

[14] Devi SA, Devi SS (2016). Numerical investigation of hydromagnetic hybrid Cu–Al_2_O_3_/water nanofluid flow over a permeable stretching sheet with suction. Int. J. Nonlin. Sci. Numer. Simul..

[15] Acharya N, Mabood F (2021). On the hydrothermal features of radiative Fe_3_O_4_–graphene hybrid nanofluid flow over a slippery bended surface with heat source/sink. J. Therm. Anal. Calorim..

[16] Zainal, N.A., Nazar, R., Naganthran, K. & Pop, I. Unsteady EMHD stagnation point flow over a stretching/shrinking sheet in a hybrid Al_2_O_3_-Cu/H_2_O nanofluid. *Int. Comm. Heat Mass Transf.* **123**, 105205 (2021).

[17] Zainal NA, Nazar R, Naganthran K, Pop I (2020). Unsteady three-dimensional MHD nonaxisymmetric homann stagnation point flow of a hybrid nanofluid with stability analysis. Mathematics.

[18] Khashi'ie NS, Arifin NM, Pop I, Nazar R (2021). Dual solutions of bioconvection hybrid nanofluid flow due to gyrotactic microorganisms towards a vertical plate. Chinese J. Phys..

[19] Khashi’ie, N.S., Hafidzuddin, E.H., Arifin, N.M. & Wahi, N. Stagnation point flow of hybrid nanofluid over a permeable vertical stretching/shrinking cylinder with thermal stratification effect. *CFD Lett.* **12**(2), 80–94 (2020).

[20] Khashi'ie NS, Arifin NM, Pop I, Nazar R (2020). Melting heat transfer in hybrid nanofluid flow along a moving surface. J. Therm. Anal. Calorim..

[21] Khan, U., Waini, I., Ishak, A. & Pop, I. Unsteady hybrid nanofluid flow over a radially permeable shrinking/stretching surface. *J. Mol. Liq.* **331**, 115752 (2021).

[22] Acharya N, Bag R, Kundu PK (2019). Influence of Hall current on radiative nanofluid flow over a spinning disk: a hybrid approach. Physica E Low. Dimens. Syst. Nanostruct..

[23] Acharya N, Maity S, Kundu PK (2020). Influence of inclined magnetic field on the flow of condensed nanomaterial over a slippery surface: the hybrid visualization. Appl. Nanosci..

[24] Acharya N, Bag R, Kundu PK (2020). On the impact of nonlinear thermal radiation on magnetized hybrid condensed nanofluid flow over a permeable texture. Appl. Nanosci..

[25] Wahid, N.S., Arifin, N.M., Khashi’ie, N.S. & Pop, I. Hybrid Nanofluid Slip Flow over an Exponentially Stretching/Shrinking Permeable Sheet with Heat Generation. *Mathematics* **9**(1), 30 (2021).

[26] Wahid, N.S., Arifin, N.M., Khashi’ie, N.S., Pop, I., Bachok, N. & Hafidzuddin, M.E. Flow and heat transfer of hybrid nanofluid induced by an exponentially stretching/shrinking curved surface. *Case Studies Therm. Eng.* **25**, 100982 (2021).

[27] Jackson JD (1963). A study of squeezing flow. Appl. Sci. Res..

[28] Stefen, M.J. Versuch Uber die scheinbare adhesion. Sitzungsberichte der Akademie der Wissenschaften in Wien. *Mathematik-Naturwissen* **69**, 713–21 (1874).

[29] Verma RL (1981). A numerical solution for squeezing flow between parallel channels. Wear.

[30] Singh P, Radhakrishnan V, Narayan KA (1990). Squeezing flow between parallel plates. Ingenieur-Archiv..

[31] Hamza EA (1999). Suction and injection effects on a similar flow between parallel plates. J. Phys. D-Appl. Phys..

[32] Ahmad S, Khan MI, Hayat T, Khan MI, Alsaedi A (2018). Entropy generation optimization and unsteady squeezing flow of viscous fluid with five different shapes of nanoparticles. Colloid Surf. A-Physicochem. Eng. Asp..

[33] Shah, R.A., Khan, A. & Shuaib, M. Analysis of squeezing flow of a viscous fluid between corotating discs with Soret and Dufour effects. *Heat Transf. Res.* **49**(11), (2018).

[34] Khan, M.I., Ahmad, S., Hayat, T. & Alsaedi, A. Entropy generation and activation energy impact on radiative flow of viscous fluid in presence of binary chemical reaction. *Int. J. Chem. React. Eng.* **16**(9), (2018).

[35] Magalakwe G, Lekoko ML, Modise K, Khalique CM (2019). Lie group analysis for MHD squeezing flow of viscous fluid saturated in porous media. Alexandria Eng. J..

[36] Basha H (2020). A generalized perspective of magnetized radiative squeezed flow of viscous fluid between two parallel disks with suction and blowing. Heat Transf..

[37] Raees A, Xu H, Liao SJ (2015). Unsteady mixed nano-bioconvection flow in a horizontal channel with its upper plate expanding or contracting. Int. J. Heat Mass Transf..

[38] Hayat T, Muhammad T, Qayyum A, Alsaedi A, Mustafa M (2016). On squeezing flow of nanofluid in the presence of magnetic field effects. J. Mol. Liq..

[39] Hayat, T., Sajjad, R, Alsaedi, A., Muhammad, T. & Ellahi, R. On squeezed flow of couple stress nanofluid between two parallel plates. *Results Phys.* **7**, 553–61 (2017).

[40] Acharya N, Bag R, Kundu PK (2021). Unsteady bioconvective squeezing flow with higher-order chemical reaction and second-order slip effects. Heat Transfer.

[41] Salehi, S., Nori, A., Hosseinzadeh, K. & Ganji, D.D. Hydrothermal analysis of MHD squeezing mixture fluid suspended by hybrid nanoparticles between two parallel plates, *Case Stud. Therm. Eng.* **21** 100650 (2020).

[42] Acharya N (2020). On the flow patterns and thermal behaviour of hybrid nanofluid flow inside a microchannel in presence of radiative solar energy. J. Therm. Anal. Calorim..

[43] Ikram MD, Asjad MI, Akgül A, Baleanu D (2021). Effects of hybrid nanofluid on novel fractional model of heat transfer flow between two parallel plates. Alexandria Eng. J..

[44] Islam, S., Khan, A., Deebani, W., Bonyah, E., Alreshidi, N.A. & Shah, Z. Influences of Hall current and radiation on MHD micropolar non-Newtonian hybrid nanofluid flow between two surfaces, *AIP Adv*. **10**(5), 055015 (2020).

[45] Oztop HF, Abu-Nada E (2008). Numerical study of natural convection in partially heated rectangular enclosures filled with nanofluids. Int. J. Heat Fluid Flow.

[46] Waini, I., Ishak, A. & Pop, I. MHD flow and heat transfer of a hybrid nanofluid past a permeable stretching/shrinking wedge. *Appl. Math. Mech.* (*Eng. Ed.*) **41**(3) 507–20 (2020).

[47] Verma, V.K. & Mondal, S. A brief review of numerical methods for heat and mass transfer of Casson fluids. *Partial Diff. Eqn. Appl. Math.* **3**, 100034 (2021).

[48] Rai, N. & Mondal, S. Spectral methods to solve nonlinear problems: A review. *Partial Diff. Eqn. Appl. Math.* **4**, 100043 (2021).

[49] Oyelakin IS, Mondal S, Sibanda P, Motsa SS (2019). A multi-domain bivariate approach for mixed convection in a Casson nanofluid with heat generation. Walailak J. Sci. Tech..

